# Status of home-based secondhand smoke exposure among children and its association with health risks in Japan

**DOI:** 10.1016/j.pmedr.2023.102585

**Published:** 2024-01-02

**Authors:** Masako Yamada, Minato Nakazawa

**Affiliations:** a Kobe Graduate School of Health Sciences, Japan; b Kobe City College of Nursing, Japan

**Keywords:** Secondhand smoke exposure, Pediatric health risk, Heated tobacco products, E-Cigarette, Maternal perception, Home-based exposure, Tobacco, Family smoking

## Abstract

**Objectives:**

In Japan, secondhand smoke (SHS) exposure in children has changed with increased heated tobacco products (HTPs) and electronic cigarettes (e-cigs) use. We clarified the status of home-based SHS exposure among children, including HTPs and e-cigs, as well as maternal perceptions on SHS avoidance and examined its association with health risks.

**Methods:**

This cross-sectional questionnaire-survey-based study included 379 mothers who were raising children aged < 5 years.

**Results:**

We found that 31.1 % of the children had home-based SHS exposure, with widespread HTP and e-cig use among smoking mothers and family members (74.2 % and 67.0 %, respectively). Significantly (*p* < 0.05), HTP and cigarettes were used in the kitchen and gardens/balconies, and maternal perceptions of child SHS-exposure avoidance were lower among smokers than non-smokers. Non-smoking mothers also had low perceptions of smoking on balconies and having immediate post-smoking contact with their children (27.6 % and 27.9 %, respectively). Most non-smoking mothers (76.4 %) reported that they do not encourage family members to quit smoking. The structural equation model showed that the presence of smoking at home and low maternal perceptions was correlated with SHS-related child health risks, including respiratory diseases, otitis media, and dental caries.

**Conclusions:**

HTP and e-cig use in the household has been rapidly increasing even among mothers and family members, and the presence of smokers at home and low maternal perceptions may be associated with child health risks. Increasing the maternal awareness of the child health risks of home-based SHS, including HTPs and e-cigs, along with continued smoking-cessation support involving mothers and families, is indispensable.

## Highlights

• SHS exposure in children has significantly changed recently in Japan.
• The use of HTPs and e-cigs in the household with children has been rapidly increasing.
• Smokers at home and low maternal perceptions may be associated with child health risks.
• It is important to raise awareness of SHS avoidance among mothers, including non-smokers.

## 1 Introduction

Secondhand smoke (SHS) exposure is associated with high morbidity and mortality rates. Globally, 28 % of SHS-exposure-related deaths occur in children (Oberg et al., 2011). Owing to their higher breathing rates, greater lung surface area (National Cancer Institute, 1999), and the developmental processes in their bodies, children are particularly vulnerable to SHS (Centers for Disease Control and Prevention (US) (2014)) and incur an increased risk of acute respiratory infections, such as pneumonia and bronchitis, middle ear infections, more frequent and severe asthma, respiratory symptoms, and slowed lung growth (Centers for Disease Control and Prevention (US) (2014)). Wheezing, coughing, and dyspnea are common in children with SHS (Centers for Disease Control and Prevention (US) (2006)).

In Japan, the circumstances surrounding SHS have significantly changed recently. By April 2020, smoke-free regulations had been enforced in public areas to minimize the negative impact of SHS (Ministry of Health, Labour, and Welfare, 2020). However, no laws or penalties prohibit smoking in households or cars with children, even though some overseas countries prohibit parents from smoking in cars with children. Moreover, electronic cigarettes (e-cigs) are considered “tobacco-like products” and are not subject to regulation. Therefore, family members, especially children, may have SHS exposure if their parents or family members smoke.

Heated tobacco products (HTP) were launched in Japan in 2014 and have since been promoted by the tobacco industry as safer alternatives to combustible cigarettes (Mallock et al., 2019, Simonavicius et al., 2019). More recently, HTP use has been rapidly increasing, with a tendency among younger and more affluent people to use HTP (Hori et al., 2021, Igarashi et al., 2021). Furthermore, the use of e-cigs, which are popular mainly in Europe (Bandi et al., 2021, Bennett et al., 2022, Kapan et al., 2020, Tattan-Birch et al., 2023) and the United States (Bandi et al., 2021, Bennett et al., 2022, Kapan et al., 2020, Tattan-Birch et al., 2023), is widespread among younger people in the child-rearing generation. E-cigs that do not contain nicotine and HTPs have been widely popular in Japanese marketplaces since the sale of those containing nicotine has been banned since 2010 by the Pharmaceutical Affairs Act. Aerosols of HTPs and e-cigs contain carcinogens and toxic substances, such as formaldehyde, acetaldehyde, and acrolein (Bekki et al., 2017, Bekki et al., 2014). A recent study showed similar incidences of asthma attacks and chest pain from secondhand exposure to HTP-generated aerosols or cigarette smoke among individuals aged 15–73 years (Imura and Tabuchi, 2021). Moreover, 37 % of adults with SHS exposure to HTP experienced at least one symptom, with the most common complaint being a generalized feeling of illness, followed by eye discomfort and sore throat (Imura and Tabuchi, 2021, Tabuchi et al., 2018). More importantly, maternal HTP smoking during pregnancy has been reported to be associated with increased risk for small for gestational age and allergy in the offspring (Hosokawa et al., 2022, Zaitsu et al., 2023). In addition, a previous study found a link between secondhand vape exposure and an increased risk of shortness of breath and bronchitic symptoms in young adults (Islam et al., 2022). However, no study has examined the health effects of home-based exposure to SHS, including HTP and e-cigs, in children. Generally, children are unable to control their environment and, thus, cannot take measures to prevent SHS exposure. Therefore, the strategies that parents or family members employ play a key role in reducing their children’s exposure to SHS (Jeong et al., 2021, Zheng et al., 2017). Particularly, mothers spend more time with their children and should be empowered to protect them from SHS exposure.

This study aimed to clarify the status of home-based SHS exposure among children, including HTPs and e-cigs, as well as maternal perceptions of child SHS avoidance and to examine its association with health risks.

## 2 Material and methods

### 2.1 Study design

This cross-sectional study was conducted in February 2022. The sample size was calculated based on Slovin’s Formula, and 400 mothers who were at least 20 years old (the minimum age for purchasing cigarettes) who were raising children aged < 5 years were recruited from a large research panel compiled by a major Japanese online research agency – Rakuten Insight. Based on age and region of residence, the participants were assigned in the same proportion as that of the stratified population in the Japanese census data. After providing written informed consent, the participants underwent eligibility screening and completed an online questionnaire survey. The study involving human participants was reviewed and approved by the Ethical Review Committee of Kobe University Graduate School of Health Sciences (No. 1040-1).

### 2.2 Questionnaire

Participant characteristics included age, marital status, education level, occupation, annual household income, number of children, spousal cooperation in childcare, social support in childcare, and well-being, as assessed based on the World Health Organization-5 (WHO-5) score, Japanese version (Awata, 2002). The characteristics of children included the child’s sex, youngest child’s age (if having two or more children), smokers at home, daily childcare, gestational week at birth, weight at birth, height at birth, and child symptoms with clinical diagnosis, such as respiratory diseases (pneumonia, bronchitis, asthma, and asthmatic bronchitis), allergic diseases (allergic rhinitis and atopic dermatitis), otitis media, and dental caries (WHO, 2023). The smoking status of mothers and family members was categorized as never, former, and daily smokers, defined as someone who had smoked more than 100 cigarettes in their lifetime, smoked more than 6 months ago, and had smoked in the last 28 days, respectively (MHLW, 2019). Participants were asked to report the type of tobacco they smoked daily, such as combustible cigs and/or HTPs and/or e-cigs. Regarding combustible cigs (including hand-rolled or little cigarettes), we defined current combustible cigarette smokers as those smoking any number of combustible cigarettes per day. Regarding HTPs, we asked the participants about the number of times they smoked any of the different types of HTPs such as Ploom Tech, IQOS, glo, per day during the survey. We defined current HTP users as those smoking any number of HTPs per day. Regarding e-cigs, we asked the participants about the number of times per day they smoked any of the different types of e-cigs and whether or not they contained nicotine, also known as vapes, during the survey. We defined current e-cig users as smoking any number of e-cigs daily. The user groups were defined as exclusive combustible cigarette smokers, exclusive HTP users, exclusive e-cig users, and any combination of two or three of these products. Furthermore, smokers were asked where they smoked or used the products at home (garden/balcony, car, own room, kitchen, bathroom, no use at home, or anywhere at home). Maternal perceptions on avoidance of SHS exposure in children were determined using a 5-point scale, from “Strongly Disagree (0)” to “Strongly Agree (4),” which was developed with reference to previous studies (Keiko, 2014), and a preliminary survey was conducted with 29 participants to determine whether the internal validity was adequate. Cronbach’s alpha for internal validity was 0.891.

### 2.3 Definitions of child health risks related to SHS and exposure variables

For the primary outcome of child health risks related to SHS, we asked the participants whether or not their children had ever been clinically diagnosed by the physicians with any of the following conditions: respiratory diseases (pneumonia (yes/no), bronchial asthma (yes/no), and asthmatic bronchitis (yes/no)), allergic diseases (allergic rhinitis (yes/no) and atopic dermatitis (yes/no)), otitis media (yes/no), and dental caries (yes/no).

Exposure variables were whether or not there were smokers, including HTPs/e-cigs (mothers and/or family members) at home.

### 2.4 Statistical analysis

Smoking places according to cigarette types, maternal perceptions on avoidance of child SHS exposure, and child symptoms with a clinical diagnosis were examined using the chi-square or Fisher’s exact test. The mean for the total score of maternal perceptions on child SHS was calculated using the unpaired Student’s *t*-test and the Mann–Whitney *U* test. Finally, structural equation modeling was applied to demonstrate the overall relationship. The degree of fit of the hypothetical model to the data was calculated using the goodness-of-fit index (GFI), adjusted GFI, and root-mean-square error of approximation. All statistical analyses were performed using IBM SPSS for Windows, version 26 and SPSS AMOS version 26 (IBM Corp., Armonk, New York, USA). In all cases, statistical significance was set at *p* < 0.05.

## 3 Results

### 3.1 Maternal characteristics

Overall, 400 mothers participated in an online survey. After excluding responses with missing values or outliers, 379 respondents were enrolled in this study. Table 1 shows the characteristics of the mothers (age, mean ± SD, 34.5 ± 5.0 years). Most respondents (64.6 %) were aged 30–39 years old. None of the mothers was older than 50 years. Most respondents (95.3 %) were married, and the most common educational level was university or higher (45.9 %), followed by college/technical school (32.5 %) and high school or lower (21.6 %). Approximately half of the respondents (57.0 %) were employed or self-employed. The annual household income was < 3 million yen (13.2 %), 3–6 million yen (42.2 %), and > 6 million yen (44.6 %). The mean (±SD) number of children was 1.7 ± 0.8, and most of the respondents had one child (44.6 %) or two children (42.2 %). Regarding childcare, 333 (87.9 %) and 294 (77.6 %) respondents had their partner’s cooperation and social support, respectively. Furthermore, according to respondents’ WHO-5 score, 203 (53.6 %) had low well-being.

**Table 1:** Participant descriptive statistics among 379 mothers in Japan, 2022.

(n = 379)			n	(%)
	Age (years)			
		mean ± SD	34.5 ± 5.0	
		20–29	63	(16.6)
		30–39	245	(64.6)
		40–49	71	(18.7)
	Marital status			
		Married	361	(95.3)
		Never married/Divorced/Widowed	18	(4.7)
	Education level			
		High school or below	82	(21.6)
		College/Technical school	123	(32.5)
		University or above	174	(45.9)
	Occupation			
		Currently unemployed	163	(43.0)
		Employed/Self-employed	216	(57.0)
	Annual household income			
		<3 million yen	50	(13.2)
		3–6 million yen	160	(42.2)
		>6 million yen	169	(44.6)
	No. of children			
		mean ± SD	1.7 ± 0.8	
		1	169	(44.6)
		2	160	(42.2)
		>3	50	(10.6)
	Spousal cooperation in childcare			
		Yes	333	(87.9)
		No/No partner	46	(12.1)
	Social support in childcare			
		Yes	294	(77.6)
		No	85	(22.4)
	WHO-5 score			
		Low well-being	203	(53.6)

SD, standard deviation; WHO, World Health Organization

### 3.2 Children’s characteristics

Table 2 presents the children’s characteristics. Among the child participants, 174 (45.9 %) and 205 (54.1 %) were boys and girls, respectively. The mean (±SD) age of children was 2.5 (±1.6) years. Overall, 118 (31.1 %) children had smokers in their family, 201 (53.0 %) used childcare facilities during the day, 335 (88.4 %) were born full-term, and 349 (92.1 %) had normal birth weight (2500–3999 g). The children in the pediatric cohort were clinically diagnosed with allergic diseases (15.0 %), respiratory diseases (5.8 %), otitis media (5.5 %), and dental caries (4.0 %).

**Table 2:** Participant descriptive statistics among 379 children in Japan, 2022.

(n = 379)			n	(%)
	Sex			
		Boys	174	(45.9)
		Girls	205	(54.1)
	Age (years)			
		mean ± SD	2.5 ± 1.6	
		0	67	(17.7)
		1	103	(27.2)
		2	76	(20.1)
		3	52	(13.7)
		4	39	(10.3)
		5	42	(11.1)
	Smokers at home			
		Yes	118	(31.1)
		No	261	(68.9)
	Daily childcare			
		Mother/Father	229	(60.4)
		Grandmother/Grandfather	37	(9.8)
		Childcare facilities	201	(53.0)
	Gestational week at birth			
		<37	37	(9.8)
		37–41	335	(88.4)
		<42	7	(1.8)
	Weight at birth			
		<1500 g	1	(0.3)
		1500–2499 g	26	(6.9)
		2500–3999 g	349	(92.1)
		>4000 g	3	(0.8)
	Symptoms with a clinical diagnosis			
		Allergic diseases	57	(15.0)
		Respiratory diseases	22	(5.8)
		Otitis media	21	(5.5)
		Dental caries	15	(4.0)

* Allergic diseases refer to atopic dermatitis and allergic rhinitis.

* Respiratory diseases refer to pneumonia, bronchitis, asthma, and asthmatic bronchitis.

### 3.3 Smoking status of mothers and family members

Table 3 shows the smoking habits of mothers and family members; 31 (8.2 %) mothers and 109 (28.8 %) family members smoked daily. Table 4 shows the cigarette typeof the smoking mothers and family members. Among the smoking mothers, the total percentage of combustible cigarette, HTP and e-cig use was 51.6 %, 64.5 %, and 9.7 % respectively. Among the smoking family members, the total percentage of combustible cigarette, HTP and e-cig use was 51.4 %, 55.0 %, and 11.9 % respectively. Table 5 shows smoking places based on cigarette type. Among mothers and family members, respectively, the most common place for smoking combustible cigarettes was the “garden or balcony” (68.8 % and 66.1 %), followed by “in the car” (31.2 % and 17.9 %) and “in the kitchen” (25.0 % and 23.2 %). However, among mothers and family members, the most common place where HTPs or e-cig users smoked was “in the kitchen” (56.5 % and 39.7 %)) and “in the car” (21.7 % and 17.8 %), respectively. Moreover, 8.7 % and 8.2 % of mothers and family members, respectively, reported that they smoked HTPs or e-cigs “anywhere at home.” Mothers and family members were more likely to smoke combustible cigarettes in the garden or on the balcony (*p* = 0.037, 0.018) and were more likely to use HTPs or e-cigs in the kitchen (*p* = 0.051, 0.047).

**Table 3:** Distribution of smoking habits of mothers and family members in Japan, 2022.

			Family members							
			Daily		Former		Never			
(n = 379)			n	(%)	n	(%)	n	(%)	Total	
Mothers										
	Daily		22	(71.0)	2	(6.5)	7	(22.6)	31	(8.2)
	Former		28	(38.4)	11	(15.1)	34	(46.0)	73	(19.3)
	Never		59	(21.5)	24	(8.7)	192	(69.8)	275	(72.6)
	Total		109	(28.8)	37	(9.8)	233	(61.5)	379	(100.0)

**Table 4:** Distribution of cigarette type used by the smoking mothers and family members in Japan, 2022.

	Mothers (n = 31)		Family members (n = 119)	
	n	(%)	n	(%)
Combustible cigs	16	(51.6)	56	(51.4)
HTPs	20	(64.5)	60	(55.0)
E-cigs	3	(9.7)	13	(11.9)

**Table 5:** Distribution of smoking places among smokers by cigarette type in Japan, 2022.

	Mothers (n = 31)						Family members (n = 119)				
	Combustible cigs.		HTPs/E-cigs.		p-value		Combustible cigs.		HTPs/E-cigs.		
	n	(%)	n	(%)			n	(%)	n	(%)	p-value
In any place below	16	(100.0)	23	(100.0)			56	(100.0)	73	(100.0)	
No use at home	0	(0.0)	2	(8.7)	0.236		5	(8.9)	6	(8.2)	0.886
Garden/Balcony	11	(68.8)	8	(34.8)	0.037		37	(66.1)	33	(45.2)	0.018
Car	5	(31.2)	5	(21.7)	0.503		10	(17.9)	13	(17.8)	0.994
Own room	2	(12.5)	1	(4.3)	0.347		2	(3.6)	6	(8.2)	0.278
Kitchen	4	(25.0)	13	(56.5)	0.051		13	(23.2)	29	(39.7)	0.047
Bathroom	0	(0.0)	0	(0.0)	–		1	(1.8)	3	(4.1)	0.450
Anywhere at home	0	(0.0)	2	(8.7)	0.226		2	(3.6)	6	(8.2)	0.278

### 3.4 Maternal perceptions on child SHS-exposure avoidance

Table 6 shows the maternal perceptions of child SHS-exposure avoidance based on the mother’s smoking status. The highest and lowest scores were 40 and 7, respectively (score, mean ± SD, 22.42 ± 5.32 and 32.91 ± 6.37 for smokers and non-smokers, respectively). Significant differences were found in all items regarding maternal perceptions of child SHS exposure between smokers and non-smokers (*p* < 0.05).

**Table 6:** Maternal perceptions on child SHS avoidance by smokers and non-smokers.

			**Smokers**		**Non-smokers**		
			(n = 31)		(n = 348)		
			n	(%)	n	(%)	p-value
**Total**			22.42 ± 5.32		32.91 ± 6.37		0.000
	**If I have a family member who smokes, I should encourage them to quit smoking.**						
		Strongly agree/agree	3	(9.7)	266	(76.4)	0.000
		Strongly disagree/disagree/ neither	28	(90.3)	82	(23.6)	
	**Smoking should be prohibited indoors at home.**						
		Strongly agree/agree	17	(54.8)	324	(93.1)	0.000
		Strongly disagree/disagree/neither	14	(45.2)	24	(6.9)	
	**Smoking should be prohibited on home balconies.**						
		Strongly agree/agree	6	(19.4)	252	(72.4)	0.000
		Strongly disagree/disagree/ neither	25	(80.6)	96	(27.6)	
	**Smoking should be prohibited under the ventilation fan in the kitchen at home.**						
		Strongly agree/agree	8	(25.8)	293	(84.2)	0.000
		Strongly disagree/disagree/neither	23	(74.2)	55	(15.8)	
	**Any family member or friend who attempts to smoke in the car should be prohibited.**						
		Strongly agree/agree	13	(41.9)	292	(83.9)	0.000
		Strongly disagree/disagree/neither	18	(58.1)	56	(16.1)	
	**Any family member/friend should be prohibited from smoking near children.**						
		Strongly agree/agree	18	(58.1)	308	(88.5)	0.000
		Strongly disagree/disagree/neither	13	(41.9)	40	(11.5)	
	**Any family member/friend should be prohibited from contacting children after smoking.**						
		Strongly agree/agree	12	(38.7)	251	(72.1)	0.000
		Strongly disagree/disagree/neither	19	(61.3)	97	(27.9)	
	**When taking children, I should choose an entirely non-smoking restaurant.**						
		Strongly agree/agree	17	(54.8)	262	(75.3)	0.013
		Strongly disagree/disagree/neither	14	(45.2)	86	(24.7)	
	**When being with children, I should avoid designated smoking areas.**						
		Strongly agree/agree	16	(51.6)	272	(78.2)	0.001
		Strongly disagree/disagree/ neither	15	(48.4)	76	(21.8)	
	**I should work not to expose children to SHS where they live and play.**						
		Strongly agree/agree	18	(58.1)	275	(79.0)	0.008
		Strongly disagree/disagree/neither	13	(41.9)	73	(21.0)	

Approximately one-third of non-smoking mothers reported that smoking was allowed in home balconies (27.6 %), and smokers were allowed contact with children immediately after smoking (27.9 %). Moreover, 23.6 % of non-smoking mothers disagreed or strongly disagreed that they should encourage family members to quit smoking.

### 3.5 Child symptoms with clinical diagnosis based on the presence of smokers at home

Table 7 shows child symptoms with clinical diagnosis based on the presence of smokers at home. Children who had smokers at home were more likely to have respiratory disease, otitis media, and dental caries (*p* = 0.049, *p* = 0.008, and *p* = 0.001, respectively).

**Table 7:** Distribution of child symptoms with clinical diagnosis based on the presence of smokers at home, including HTPs/e-cigs.

		**Smokers at home**		**No smokers at home**		
		(n = 118)		(n = 261)		
		n	(%)	n	(%)	p-value
**RD**	Diagnosed	11	(9.3)	11	(4.2)	0.049
	Never	107	(90.7)	250	(95.8)	
**Allergic diseases**	Diagnosed	19	(16.1)	38	(14.6)	0.697
	Never	99	(83.9)	223	(85.4)	
**Otitis media**	Diagnosed	12	(10.2)	9	(3.4)	0.008
	Never	106	(89.8)	252	(96.6)	
**Dental caries**	Diagnosed	11	(9.3)	4	(1.5)	0.001
	Never	107	(90.7)	257	(98.5)	

HTPs, heated tobacco products; e-cigs, electronic cigarette. RD, respiratory diseases.

### 3.6 Structural equation modeling of factors that affect child health risks related to SHS

**Fig. 1:**
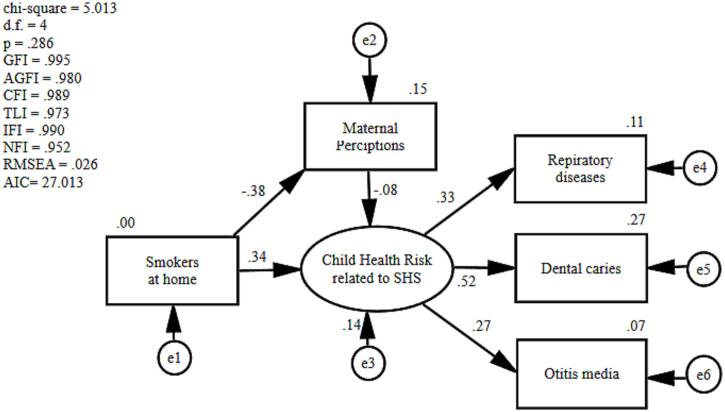
Association with child health risk related to SHS and smokers at home, including HTPs/e-cigs and maternal perception on avoidance of SHS, in Japan, 2022 SHS, secondhand smoke; HTPs, heated tobacco products; e-cigs, electronic cigarettes.

Fig. 1 shows the structural equation modeling of factors that affect children’s SHS-related health risks. In this study, smoking at home was significantly positively correlated with child SHS-related health risks, such as respiratory diseases, otitis media, and dental caries. Maternal perceptions negatively correlated with SHS-related child health risks.

## 4 Discussion

In this study, we demonstrated that several children had home-based SHS exposure, including HTPs and e-cigs, and many of those mothers and families were using HTP and e-cigs in the household, such as in the kitchen, room, car, and garden/balcony. Our findings also indicated that the presence of HTP and e-cig users at home and low maternal perceptions of child SHS avoidance may be associated with SHS-related child health risks, such as respiratory diseases, otitis media, and dental caries.

The use of HTPs and e-cigs in the household has been rapidly spreading, even among mothers and families with children. Combustible smokers may have switched to HTPs or e-cigs owing to the influence of ‘‘Point of Purchase’’ that emphasized the positive aspects, such as “less smell” and “no air pollution,” of HTP and e-cigs (Kim et al., 2020), or it could be that they used HTP indoors and combustible cigarettes outside or in public smoking areas (Kopp et al., 2018). Many studies have shown that when pregnant women, mothers, and family members smoke, children are exposed to the health effects of not only SHS but also tertiary smoking, where children inhale toxic substances on the smoker’s clothing and hair (Centers for Disease Control and Prevention (US), 2014, Centers for Disease Control and Prevention (US), 2006, Hanioka et al., 2011, Johansson et al., 2004, Sleiman et al., 2010, Strachan and Cook, 1998) In addition, the use of HTPs during pregnancy has been reported to be associated with asthma, rhinitis, conjunctivitis, atopic dermatitis, and being small for gestational age. Moreover, aerosols from HTPs spread across more than 2 m and, simultaneously, the concentration of fine particulate matter (PM2.5) at 2 m reached 100–800 μg/m^3^(n.d.); thus, the room was contaminated through secondary exposure, which was equivalent to SHS. Nicotine exposure can affect embryonic stem cell differentiation into fibroblasts, resulting in impaired lung growth and decreased lung function among children (Gibbs et al., 2016, Strzelak et al., 2018) as well as otitis media (Adair-Bischoff and Sauve, 1998, Ey et al., 1995, Teele et al., 1989). HTP and e-cig users were less likely to quit the product they used than exclusive smokers (Lee et al., 2020). Therefore, this finding suggests that quitting HTP and e-cig use among mothers and family members is beneficial in promoting child health.

Regarding maternal perceptions of child SHS avoidance, smoking mothers scored significantly lower than non-smoking mothers. The previous study reported that current smokers and those with family members who smoked or lived with them had lower risk perceptions of SHS (Junus et al., 2021). In addition, mothers with a smoking habit are more likely to be unaware of their children’s oral health, which may contribute to dental caries in their children (Watanabe et al., 2020). Furthermore, family smoking is a risk factor for the postpartum resumption of smoking or the initiation of new smoking (Taki et al., 2018; Yasuda et al., 2013). Subsequently, this study found that even non-smoking mothers, including former smokers, were tolerant of smoking in gardens/balconies and had immediate post-smoking contact with their children. Moreover, most mothers did not even encourage family members to quit smoking. A previous study found that approximately 35 % of non-smokers did not feel an aversion to cigarette smoke around people (Otake, 2014); it has also been demonstrated that modifying unhealthy behavioral habits is viewed as a five-stage process (precontemplation, contemplation, action, maintenance, and relapse), and treatment and intervention according to each stage has proven to be effective (Prochaska and DiClemente, 1983). This study found that even if the mothers did not smoke, differences were found in how they responded to smoking and secondhand smoke around their children. Therefore, enhancing skills to help smokers quit among non-smokers is also important (Otake, 2014). However, mothers who encouraged family members who were smokers around them to quit were limited. Therefore, our findings suggest that it is important to raise awareness of SHS avoidance among mothers, including non-smokers so that they would acquire the skills to help those around their children quit smoking and to prohibit or refuse smoking in places where their children live.

Limitations.

This study had some limitations. First, because this study used a cross-sectional design, we could not explain the causality between child health risk and home-based SHS exposure, including HTPs and e-cigs. Second, this study used self-reported data, and we could not accurately validate the SHS status of the children. Therefore, longitudinal cohort studies are needed to examine the health effects of SHS, including HTP and e-cigs, along with the measurement of urinary cotinine or blood levels. In addition, the participant in this study were recruited from a large research panel compiled by an online research agency, rendering the population susceptible to selection bias. Therefore, the samples were not the representative of the general population and nationally representative surveys are needed to complement data.

## 5 Conclusions

The results of this study showed that the use of HTPs and e-cigs in the household has been rapidly increasing, even among mothers and family members raising children. In addition, the presence of smokers at home, including HTP/e-cig users, along with low maternal perceptions of avoidance of SHS may be associated with child health risks. Therefore, there is an urgent need to increase maternal perceptions of home-based SHS exposure, especially from HTP and e-cig use. 10.13039/100014337Furthermore, continued support for smoking cessation by involving mothers and families is needed in various settings to avoid home-based SHS exposure in children.

## CRediT authorship contribution statement

**Masako Yamada:** . **Minato Nakazawa:** Writing – review & editing, Supervision, Investigation, Formal analysis, Conceptualization.

## Declaration of competing interest

The authors declare that they have no known competing financial interests or personal relationships that could have appeared to influence the work reported in this paper.

## Data Availability

Data will be made available on request.
